# Supplementary material for: Genome-wide analysis in the mouse embryo reveals the importance of DNA methylation for transcription integrity
Source: Nat Commun. 2020 Jun 19;11:3153. doi: 10.1038/s41467-020-16919-w (PMC7305168; doi:10.1038/s41467-020-16919-w)
Supplement: Supplementary file 1 — Supplementary Information [file 41467_2020_16919_MOESM1_ESM.pdf]

Supplementary Information

**Genome-wide analysis in the mouse embryo reveals the importance of DNA methylation for transcription integrity**

Dahlet T et al.

4 Supplementary Tables

11 Supplementary Figures

| Sample | sex | read pairs (2x75 bp) | % bisulfite conversion | median depth | CpG 8X |
|---|---|---|---|---|---|
| WT | M | 38 871 210 | 99.68 | 77X | 1 300 606 |
| WT | F | 43 050 321 | 99.69 | 50X | 1 265 422 |
| WT | F | 45 345 745 | 99.64 | 92X | 1 378 414 |
| *Dnmt1-/-* | M | 29 941 359 | 99.72 | 35X | 1 236 187 |
| *Dnmt1-/-* | F | 30 217 435 | 99.66 | 51X | 1 330 663 |
| *Dnmt1-/-* | M | 34 954 873 | 99.65 | 68X | 1 257 331 |
| | | | | | |
| WT | F | 38 276 481 | 98.53 | 75X | 1 045 177 |
| WT | M | 52 382 980 | 98.89 | 110X | 1 059 325 |
| *Dnmt3a-/+* | F | 46 831 250 | 99.75 | 90X | 1 347 352 |
| *Dnmt3a-/+* | M | 18 981 740 | 99.81 | 52X | 1 092 934 |
| *Dnmt3a-/+* | F | 41 117 131 | 99.68 | 94X | 1 017 974 |
| *Dnmt3a-/+ Dnmt3b-/+* | M | 29 391 912 | 99.88 | 65X | 1 344 204 |
| *Dnmt3a-/+ Dnmt3b-/+* | F | 28 919 109 | 99.88 | 57X | 1 304 744 |
| *Dnmt3a-/+ Dnmt3b-/+* | M | 37 617 612 | 99.75 | 102X | 1 003 420 |
| DKO | M | 43 684 564 | 99.23 | 89X | 1 062 550 |
| DKO | F | 39 043 128 | 99.24 | 119X | 947 512 |
| DKO | M | 35 233 905 | 99.79 | 56X | 1 261 249 |

**Supplementary Table 1. Sequencing statistics for RRBS experiments performed in embryos.**

| Sample | sex | read pairs (2x100 bp) | % bisulfite conversion | mean depth | CpG 5X |
|---|---|---|---|---|---|
| WT | M | 301 591 204 | 98.8 | 13.06X | 19 295 619 |
| WT | F | 298 901 743 | 98.2 | 11.30X | 18 959 550 |
| *Dnmt1-/-* | M | 295 405 005 | 99.1 | 12.31X | 19 231 967 |
| *Dnmt1-/-* | F | 325 808 699 | 97.2 | 11.12X | 19 105 488 |
| DKO | M | 317 554 047 | 99.2 | 11.88X | 19 103 578 |
| DKO | F | 332 380 126 | 99.1 | 13.24X | 19 591 334 |

**Supplementary Table 2. Sequencing statistics for WGBS experiments performed in embryos.**

| Sample | read pairs (2x75 bp) | % bisulfite conversion | median depth | CpG 8X |
|---|---|---|---|---|
| cDKO d23_no tam | 40 523 255 | 99.18 | 99X | 1 197 481 |
| cDKO d23_no tam | 45 557 733 | 99.51 | 95X | 1 398 281 |
| cDKO d23_no tam | 32 890 630 | 99.42 | 71X | 1 301 220 |
| cDKO d23_tam | 27 915 809 | 99.66 | 62X | 1 318 796 |
| cDKO d23_tam | 38 811 224 | 99.69 | 86X | 1 293 752 |
| cDKO d23_tam | 38 696 177 | 99.64 | 80X | 1 346 863 |
| cDKO d69_no tam | 43 685 660 | 99.68 | 68X | 1 266 329 |
| cDKO d69_no tam | 33 588 364 | 99.77 | 71X | 1 243 865 |
| cDKO d69_no tam | 41 697 544 | 99.81 | 93X | 1 029 797 |
| cDKO d69_tam | 34 326 404 | 99.83 | 69X | 1 374 412 |
| cDKO d69_tam | 41 142 254 | 99.83 | 87X | 1 257 422 |
| cDKO d69_tam | 42 890 920 | 99.82 | 88X | 1 340 905 |
| **Sample** | **read pairs (2x100 bp)** | **% bisulfite conversion** | **median depth** | **CpG 8X** |
| Dnmt1 cKO d5_no tam | 31 711 743 | 99.92 | 54X | 1 355 164 |
| Dnmt1 cKO d5_no tam | 19 854 418 | 99.93 | 36X | 1 511 248 |
| Dnmt1 cKO d5_no tam | 29 111 078 | 99.93 | 55X | 1 425 416 |
| Dnmt1 cKO d5_tam | 24 769 564 | 99.94 | 46X | 1 481 505 |
| Dnmt1 cKO d5_tam | 19 271 946 | 99.94 | 37X | 1 423 048 |
| Dnmt1 cKO d5_tam | 21 833 127 | 99.94 | 41X | 1 411 656 |
| Dnmt1 cKO d7_no tam | 38 951 444 | 99.93 | 61X | 1 225 672 |
| Dnmt1 cKO d7_no tam | 42 453 385 | 99.92 | 57X | 1 161 279 |
| Dnmt1 cKO d7_no tam | 26 416 927 | 99.94 | 53X | 1 359 907 |
| Dnmt1 cKO d7_tam | 37 534 599 | 99.93 | 52X | 1 288 825 |
| Dnmt1 cKO d7_tam | 26 065 819 | 99.96 | 48X | 1 473 298 |
| Dnmt1 cKO d7_tam | 24 741 169 | 99.96 | 44X | 1 469 496 |

**Supplementary Table 3. Sequencing statistics for RRBS experiments performed in MEFs.**

| Sample | sex | read pairs (2x100 bp) | % uniquely mapped reads |
|---|---|---|---|
| WT | M | 122 739 365 | 83.33 |
| WT | F | 104 213 248 | 83.04 |
| WT | F | 121 124 866 | 80.21 |
| *Dnmt1-/-* | M | 114 210 283 | 72.77 |
| *Dnmt1-/-* | F | 109 543 827 | 71.88 |
| *Dnmt1-/-* | M | 102 199 179 | 71.64 |
| | | | |
| WT | M | 87 649 101 | 82.89 |
| WT | M | 91 463 316 | 83.05 |
| *Dnmt3a-/+* | F | 76 419 000 | 82.02 |
| *Dnmt3a-/+* | F | 80 239 982 | 76.14 |
| *Dnmt3a-/+* | F | 56 665 660 | 76.88 |
| *Dnmt3a-/+* | M | 63 281 010 | 64.84 |
| DKO | M | 77 483 594 | 79.22 |
| DKO | F | 88 310 497 | 81.43 |
| DKO | F | 81 180 800 | 81.14 |
| DKO | M | 80 244 675 | 78.73 |
| DKO | M | 53 957 539 | 59.30 |
| DKO | M | 68 230 903 | 56.05 |

**Supplementary Table 4. Sequencing statistics for RNA-seq experiments performed in embryos.**

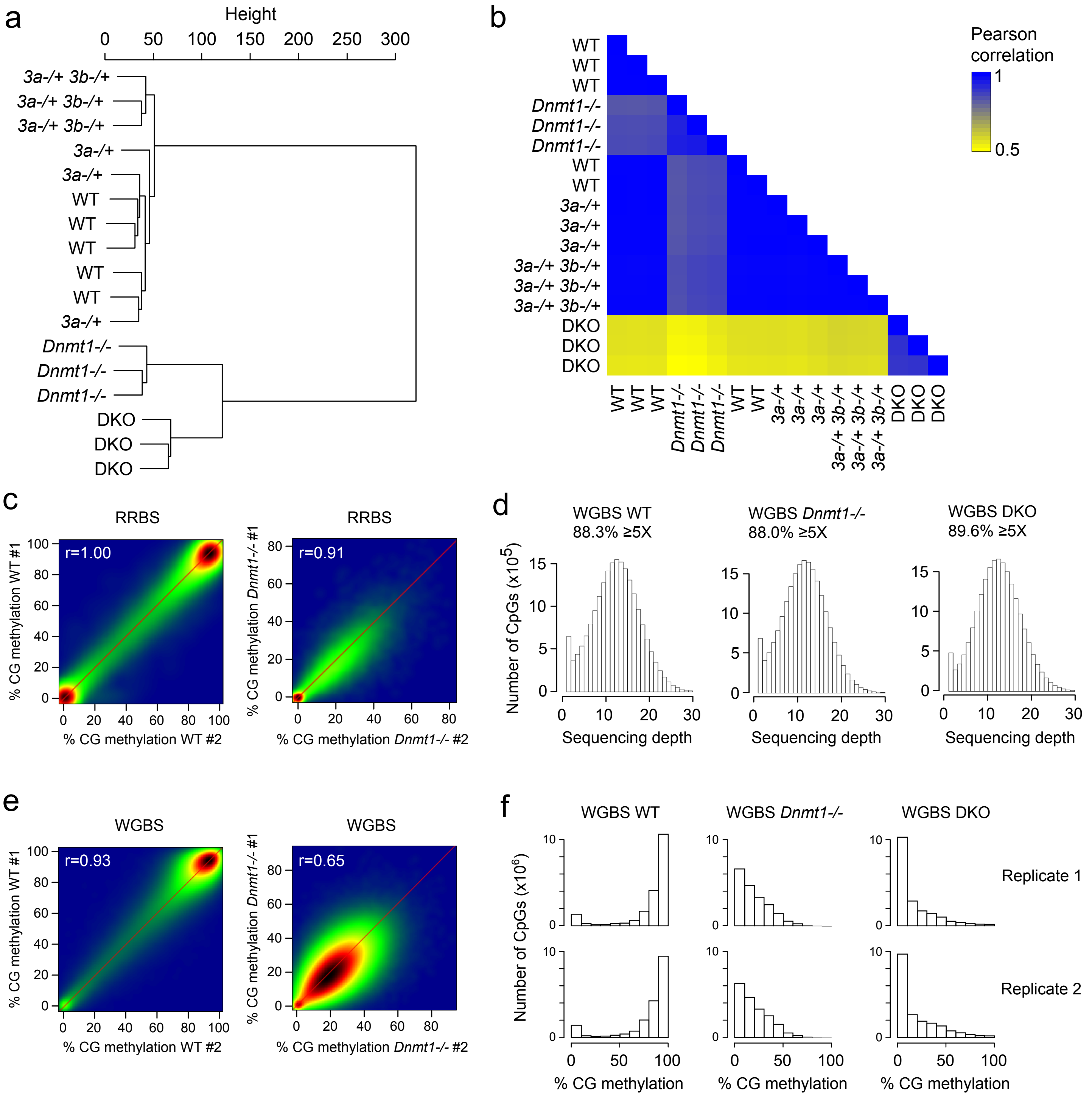


**Supplementary Figure 1. Methylome profiling of *Dnmt* mutant embryos by RRBS and WGBS. a.** Dendrogram of the hierarchical clustering of CG methylation scores in all RRBS samples. **b.** Matrix of pearson correlation coefficients of CG methylation scores in 500 bp windows between all RRBS samples. **c.** Density scatter plots showing examples of correlation of CG methylation scores in 500 bp windows between biological replicates of RRBS. The density of points increases from blue to dark red. **d.** Histograms of the sequencing depth at CGs in one replicate of WGBS for each genotype. The percentage of genomic CGs sequenced at least 5X is indicated for each sample above the graph. **e.** Correlation of CG methylation scores in 500bp windows between biological replicates of WGBS. **f.** Distribution of methylation scores at individual CGs obtained in two biological replicates of WGBS in WT, *Dnmt1-/-* and DKO embryos.

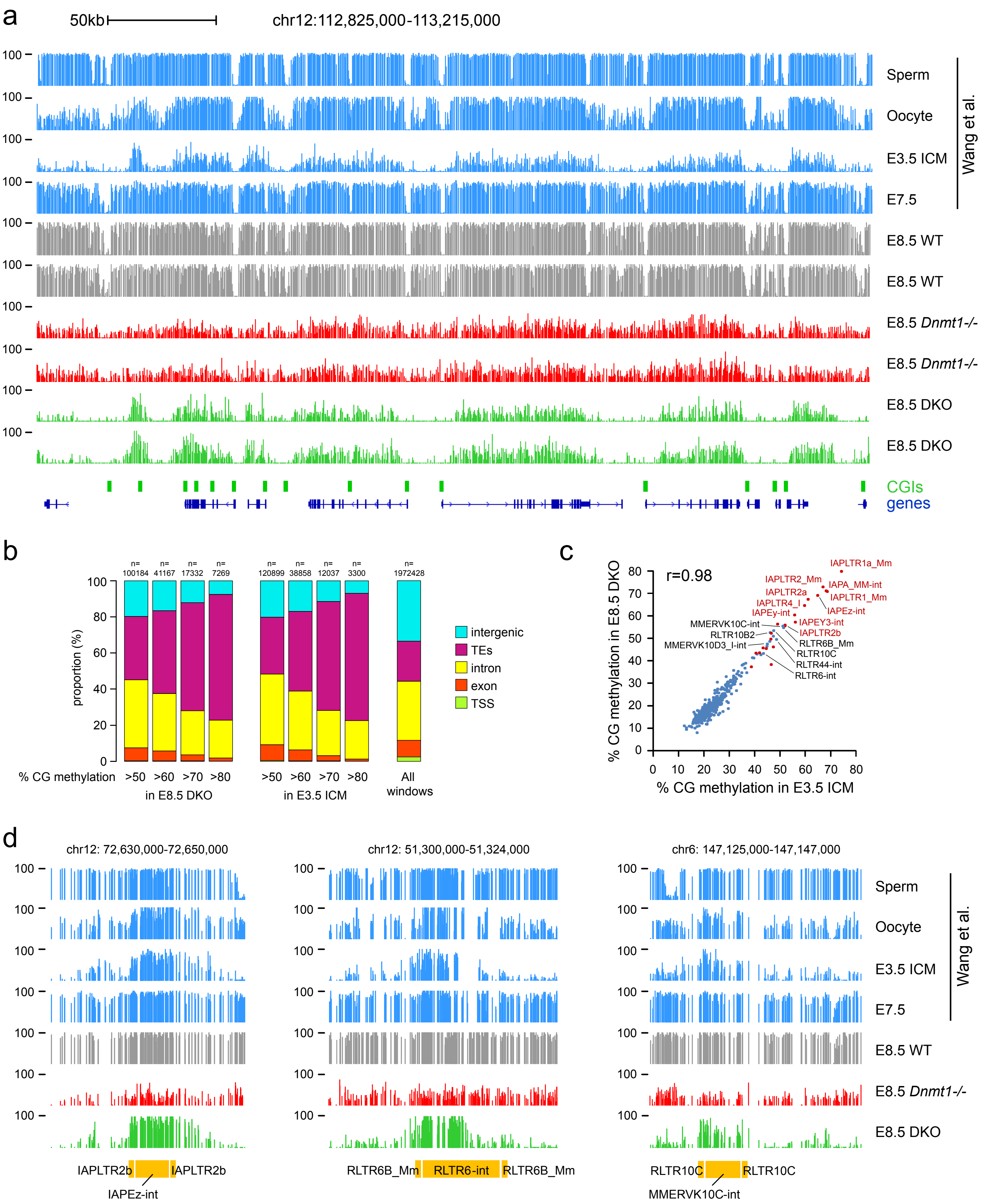


**Supplementary Figure 2. Comparison of methylomes in DKO embryos and blastocysts. a.** Example of genome browser view of WGBS methylation patterns in gametes, early embryos and E8.5 *Dnmt* mutant embryos. CpG islands (CGIs) depicted by green rectangles and RefSeq gene annotations are shown below the tracks. **b.** Genomic features associated with highly methylated sequences (1kb windows) in E8.5 DKO embryos and ICM, which illustrates that the most methylated sequences are enriched for transposable elements (TEs). **c.** Comparison of methylation levels of retrotransposon families (LTRs and LINEs) in E8.5 DKO embryos and ICM. The red dots represent IAP families. **d.** Examples of WGBS methylation profiles over transposable elements in early embryos and *Dnmt* mutant embryos. Only one WGBS replicate is shown. ERVs annotated by RepeatMasker are displayed in yellow below the tracks.

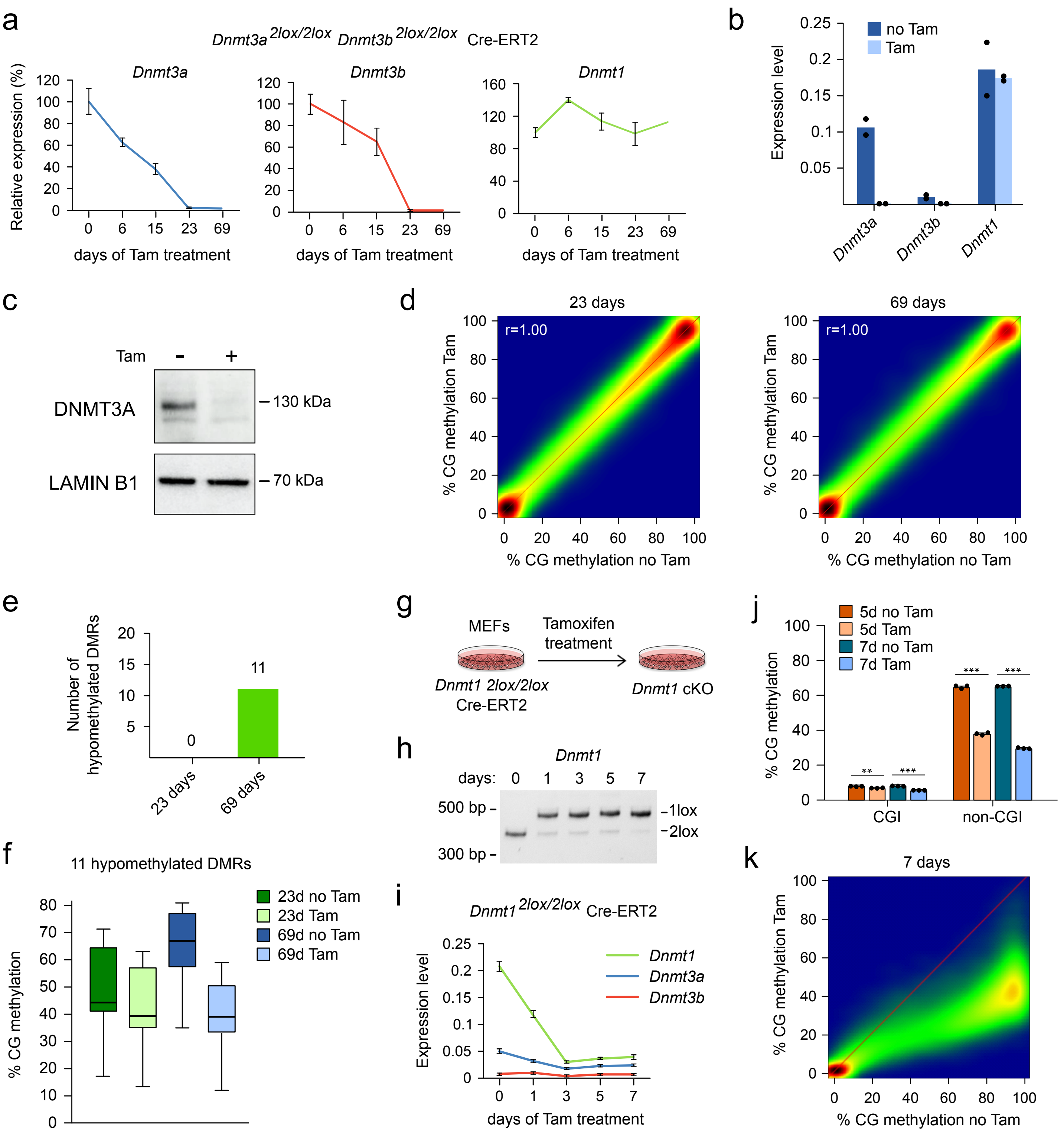


**Supplementary Figure 3. Conditional inactivation of *Dnmt1* but not *Dnmt3a/b* in MEFs results in DNA hypomethylation. a.** Kinetics of expression of *Dnmt3a/b* and *Dnmt1* by RT-qPCR in *Dnmt3a/b* cDKO fibroblasts following tamoxifen (Tam) treatment. The primers for *Dnmt3a/b* were designed in the floxed exons. The values are normalized to three housekeeping genes (*Gusb*, *Rpl13a*, *Mrpl32*) and to the day 0 (mean ± SEM, n=3 biological replicates except n=2 at day 69). **b.** Expression of *Dnmt3a/b* and *Dnmt1* measured by RT-qPCR in *Dnmt3a*$^{2lox/2lox}$*Dnmt3b*$^{2lox/2lox}$ fibroblasts treated with tamoxifen (Tam) and not treated with tamoxifen (no Tam) at 69 days of culture (mean of n=2 biological replicates). **c.** Western blot confirms the loss of DNMT3A protein in *Dnmt3a/b* cDKO fibroblasts. LAMIN B1 served as loading control. **d.** Density scatter plots of RRBS methylation scores in 500 bp windows in *Dnmt3a/b* cDKO fibroblasts treated with tamoxifen (Tam) compared to cells not treated with tamoxifen (no Tam) after 23 and 69 days of culture. **e.** Number of hypomethylated DMRs (>20% methylation loss) in *Dnmt3a/b* cDKO fibroblasts after 23 and 69 days of culture. **f.** Boxplot of CG methylation in hypomethylated DMRs (n=11) identified in *Dnmt3a/b* cDKO fibroblasts at 69 days of culture. Line, median; box limits, upper and lower quartiles; whiskers, 1.5 IQR from the quartiles. **g.** Experimental outline for generating *Dnmt1* conditional knockout (cKO) MEFs. **h.** PCR genotyping confirms the recombination of *Dnmt1* 2lox alleles in *Dnmt1* cKO MEFs. The number of days of tamoxifen treatment is indicated above the gel. The conditional inactivation was repeated three times independently. **i.** Kinetics of expression of *Dnmt1* and *Dnmt3a/b* by RT-qPCR in *Dnmt1* cKO MEFs following tamoxifen treatment (mean ± SEM, n=3 biological replicates). **j.** CG methylation levels quantified by RRBS in CpG islands (CGI) and non-CGI regions in *Dnmt1* cKO MEFs after 5 and 7 days of tamoxifen treatment (mean ± SEM, n=3 biological replicates). **: $p$<0.01; ***: $p$<0.001 (two-tailed unpaired t-test). **k.** Density scatter plot of RRBS methylation scores in 500 bp windows in *Dnmt1* cKO fibroblasts treated with tamoxifen (Tam) for 7 days compared to cells not treated with tamoxifen (no Tam). In **d**, **f**, **k**, values are the mean of n=3 biological replicates.

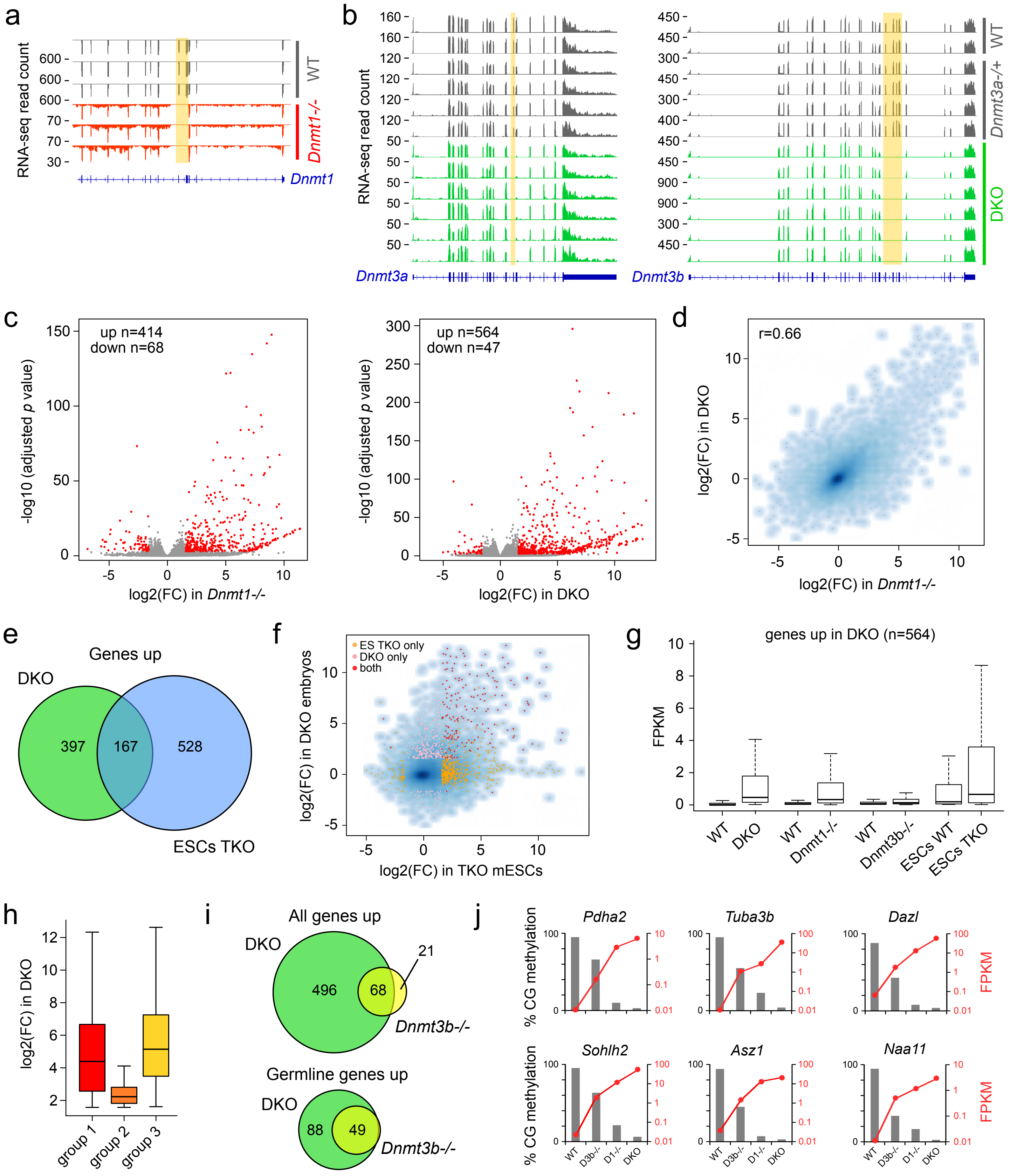


**Supplementary Figure 4. Differentially expressed genes in *Dnmt* mutant embryos. a-b.** RNA-seq validates the absence of the exons 4-5 of *Dnmt1* and catalytic exons of *Dnmt3a/b* (highlighted in yellow) in mutant embryos. **c.** Volcano plot representation of gene expression changes in *Dnmt1-/-* and DKO embryos. Red dots indicate significantly differentially expressed genes (fold change >3, adjusted *p*-value <0.001). Adjusted *p*-values were calculated by DESeq2 using a Wald test corrected for multiple testing. **d.** Comparison of gene expression changes in *Dnmt1-/-* (horizontal axis) and DKO (vertical axis) embryos. **e.** Venn diagram comparing the genes upregulated in DKO embryos and *Dnmt* triple knock-out (TKO) ES cells (Domcke et al., 2015). **f.** Comparison of gene expression changes in TKO ES cells (horizontal axis) and DKO embryos (vertical axis). Orange, pink and red dots represent genes dysregulated in TKO ES cells only, in DKO embryos only or in both. **g.** Expression level (FKPM) in *Dnmt* mutant embryos and ES cells of the genes upregulated in DKO embryos (n=564). **h.** Fold change of expression of the 3 groups of genes upregulated in DKO embryos (related to Figure 4C). Group 1: n=206; group 2: n=112; group 3: n=241. **i.** Venn diagrams comparing the genes upregulated in DKO and *Dnmt3b-/-* embryos (top), or only germline genes upregulated in DKO and *Dnmt3b-/-* embryos (bottom). **j.** Correlation between the level of promoter DNA methylation (grey bars) and expression (FPKM, red dots) for six germline genes in *Dnmt3b-/-*, *Dnmt1-/-* and DKO embryos. Boxplots: line, median; box limits, upper and lower quartiles; whiskers, 1.5 IQR from the quartiles.

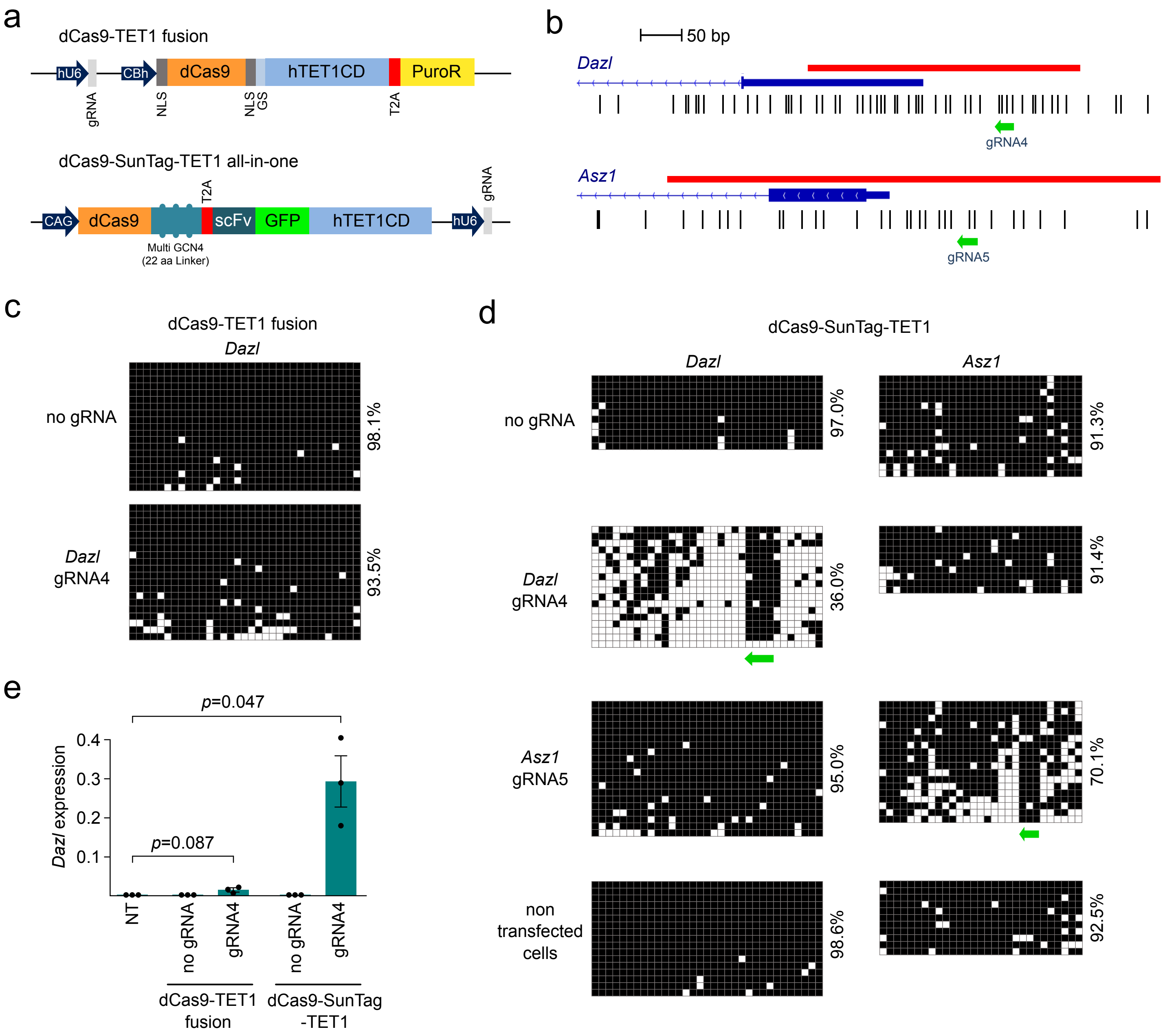


**Supplementary Figure 5. Targeted demethylation of CpG-rich promoters of germline genes in mouse fibroblasts using CRISPR-dCas9. a.** Vectors used in the experiments. We used a vector coding for dCas9 fused to the catalytic domain of the human TET1 protein (hTET1CD, top) and the all-in-one vector coding for the dCas9-Suntag-TET1 system (top) able to recruit multiple copies of hTET1CD (Morita et al., 2016). Arrows indicate promoter sequences. NLS= Nuclear Localisation Signal; GS = GS linker. **b.** Schematic representation of the *Dazl* and *Asz1* promoters. Vertical bars indicate the position of CpGs, green arrows indicate the position of gRNAs, and red bars indicate the amplicons used for bisulfite PCR. **c.** Bisulfite sequencing analysis of the *Dazl* promoter in fibroblasts transfected with the dCas9-TET1 fusion vector containing no gRNA or the *Dazl* gRNA4, indicating low demethylation efficiency. White squares represent unmethylated CpGs and black squares represent methylated CpGs. **d.** Patterns of CpG methylation of the *Dazl* and *Asz1* promoters by bisulfite sequencing analysis in fibroblasts transfected with the dCas9-SunTag-TET1 all-in-one vector containing no gRNA, the *Dazl* gRNA4, the *Asz1* gRNA5 and non-transfected cells. The green arrows indicate to CpGs contained in the gRNA sequences. **e.** Comparision of *Dazl* expression measured by RT-qPCR in fibroblasts transfected with the dCas9-TET1 fusion vector or dCas9-SunTag-TET1 vector containing no gRNA or the *Dazl* gRNA4. Expression is normalized to three housekeeping genes (*Gusb*, *Rpl13a*, *B2m*) (mean ± SEM, n=3 independent experiments). NT= non transfected cells. *p*-values: two tailed unpaired t-test.

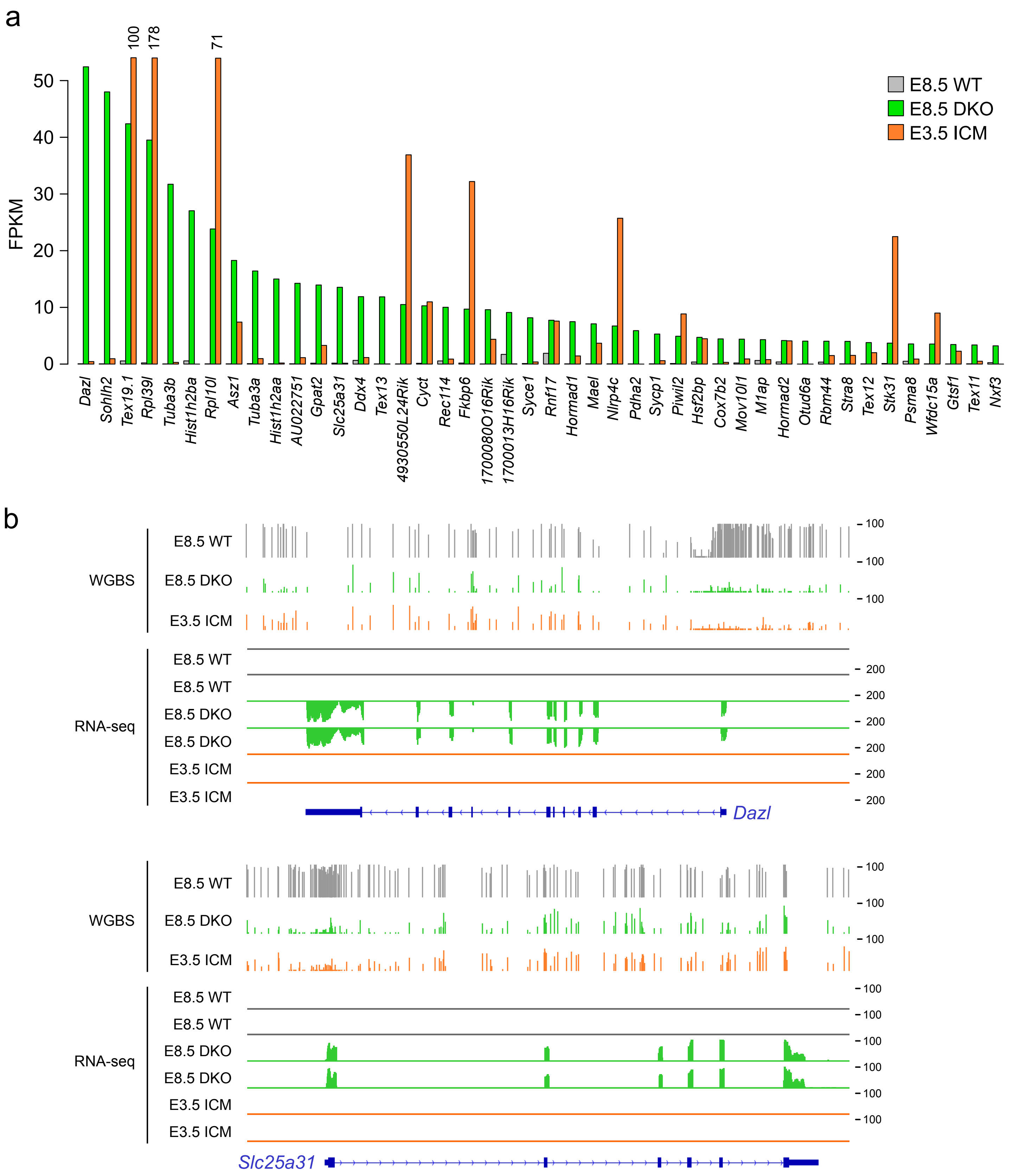


**Supplementary Figure 6. Many germline genes are derepressed in E8.5 DKO embryos but not in blastocysts. a.** Comparison of RNA-seq expression values (plotted as FPKM) in E8.5 embryos and E3.5 ICM for the 44 most derepressed germline genes in DKO embryos. **b.** Genome browser tracks showing WGBS and RNA-seq profiles of the *Dazl* and *Slc25a31* genes in E8.5 embryos and E3.5 ICM. These examples illustrate that several germline genes are not derepressed in E3.5 ICM despite similar hypomethylation than in E8.5 DKO embryos.

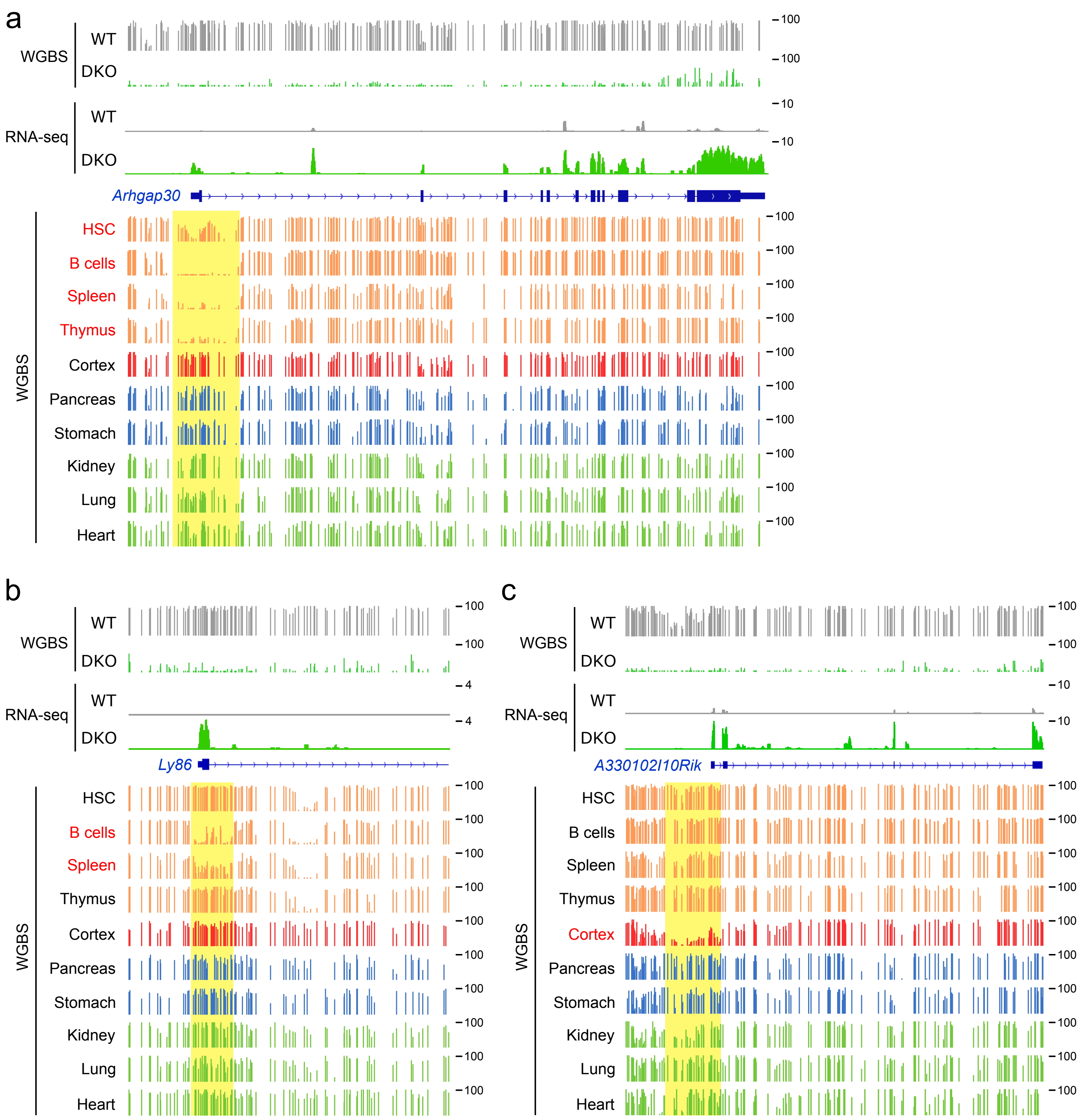


**Supplementary Figure 7. DNA methylation profiles of lineage-committed genes upregulated in DKO embryos.** Genome browser tracks of RNA-seq and WGBS methylation profiles in WT and DKO embryos, and WGBS methylation profiles in adult tissues (Cabezas-Wallscheid et al., 2014; Duncan et al., 2018; Hon et al., 2013) for the *Arhgap30* (**a**), *Ly86* (**b**) and *A330102I10Rik* (**c**) genes. For WT and DKO embryos, one replicate of RNA-seq and WGBS is shown. These genes show tissue-specific DNA hypomethylation in their promoter sequences (highlighted in yellow). The tissues or cells where the genes are expressed are written in red. HSC = Hematopoietic Stem Cells.

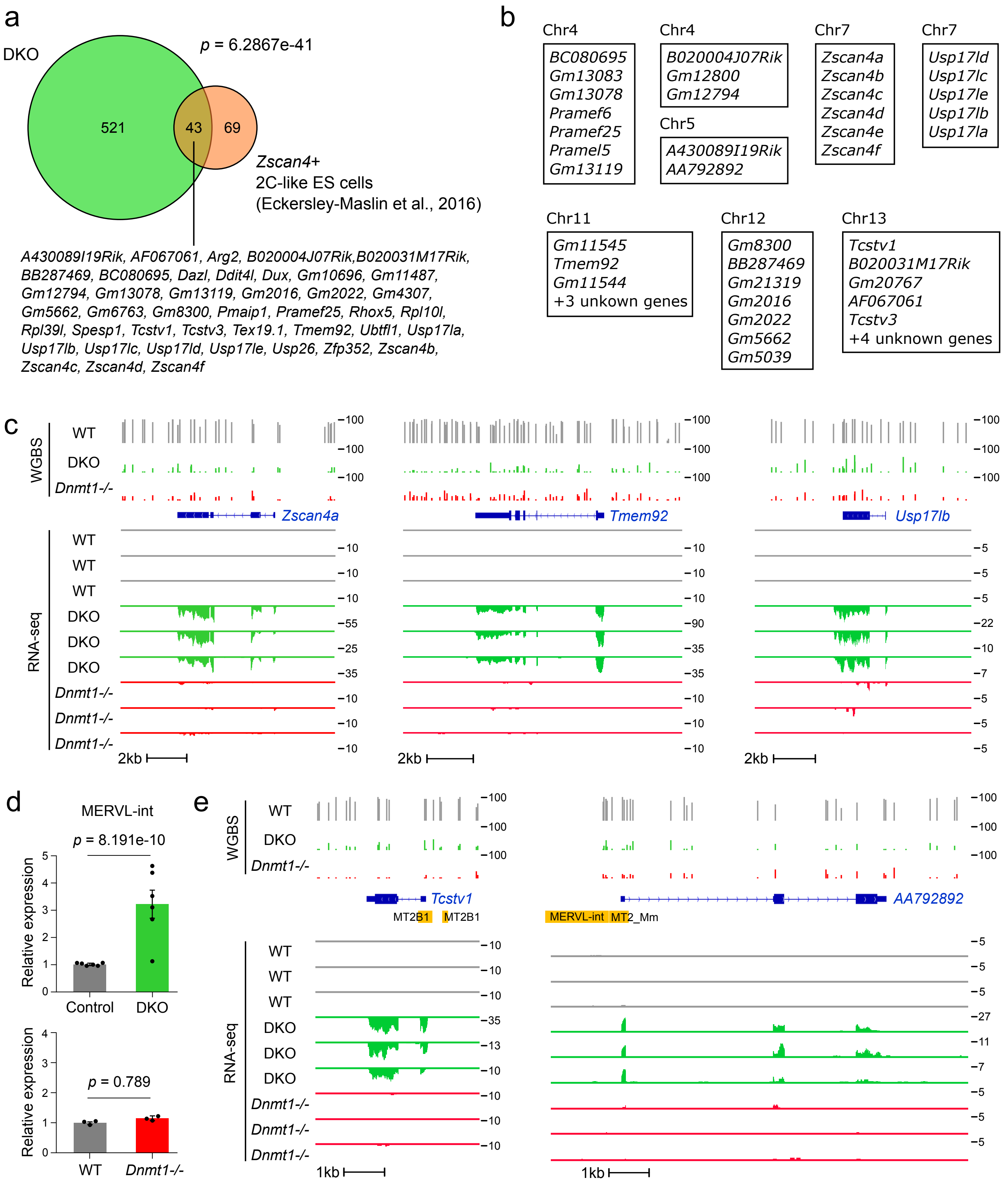


**Supplementary Figure 8. 2C-specific genes and MERVL transposons are derepressed in DKO embryos. a.** Venn diagram comparing the lists of genes upregulated in DKO embryos and in *Zscan4*+ 2C-like ES cells (Eckersley-Maslin et al., 2016). The names of the 43 common genes are indicated. The *p*-value indicates the significance of the overlap (hypergeometric test). **b.** Examples of gene clusters of 2C-specific genes derepressed in DKO embryos. **c.** Genome browser views of 2C-specific genes derepressed in DKO embryos. The top lanes show one WGBS replicate and the bottom lanes show three RNA-seq replicates in WT, DKO and *Dnmt1-/-* embryos. **d.** Expression of MERVL-int transposons (measured as RNA-seq normalized read counts in RepeatMasker MERVL-int sequences) in DKO and *Dnmt1-/-* embryos. The values are normalized to control embryos (mean ± SEM, n=6 embryos for DKO, n=3 embryos for *Dnmt1-/-*). *p*-values: adjusted *p*-values calculated by DESeq2 using a Wald test corrected for multiple testing. **e.** Examples of genes derepressed in DKO embryos that initiate from ERVL sequences. RepeatMasker annotations are shown in yellow below the gene.

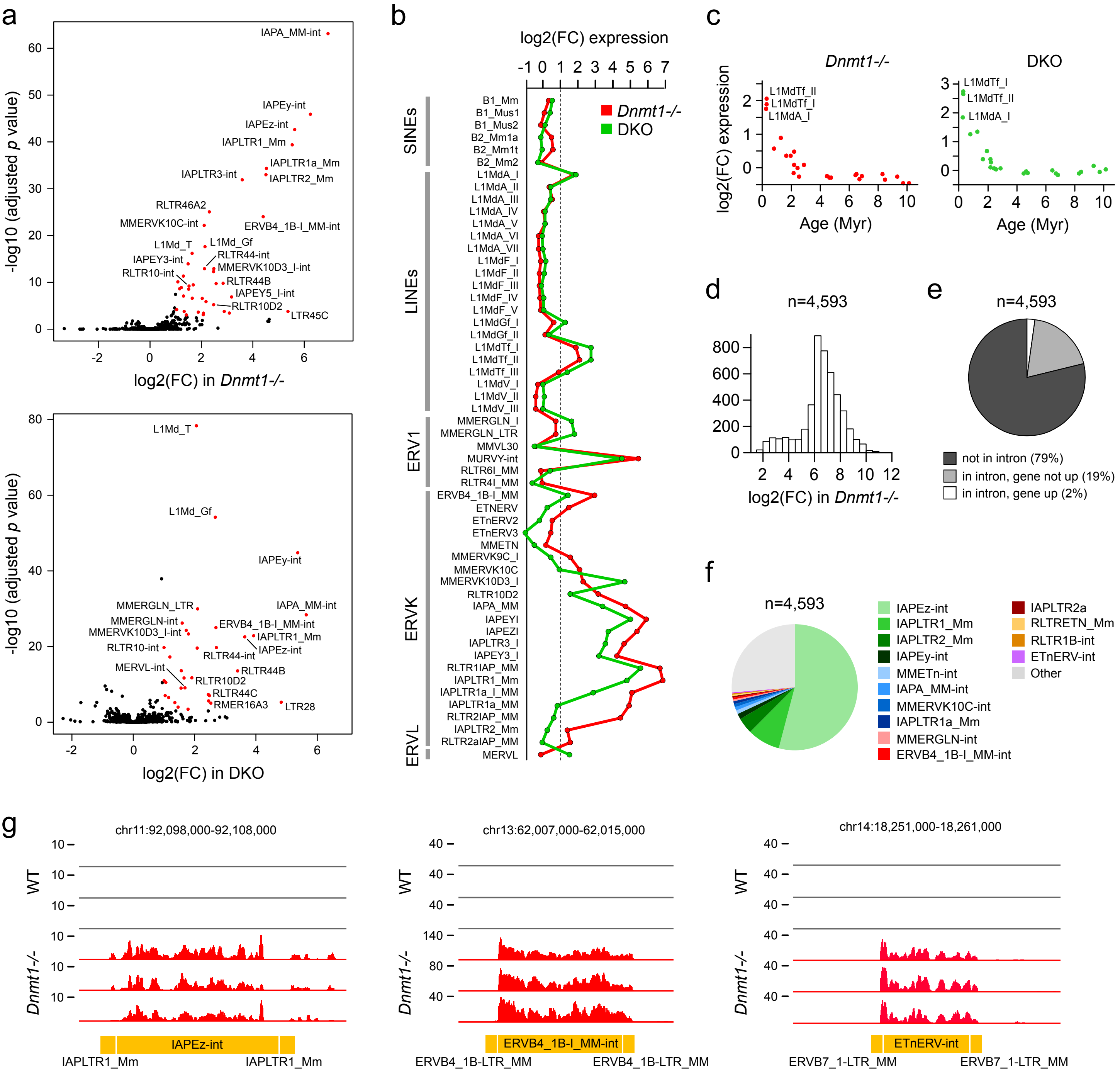


**Supplementary Figure 9. Reactivation of retrotransposons in *Dnmt* mutant embryos. a.** Volcano plot representing the changes in expression of transposon families in *Dnmt1-/-* and DKO embryos, measured by counting unique and multi-mapping RNA-seq reads in RepeatMasker annotations. Significantly upregulated transposon families (fold change > 2, adjusted *p*-value < 0.001) are highlighted in red. Adjusted *p*-values were calculated by DESeq2 using a Wald test corrected for multiple testing. **b.** Fold change of expression of SINE, LINE, ERV1, ERVK and ERVL retrotransposon families in *Dnmt1-/-* and DKO embryos, measured by mapping RNA-seq reads to Repbase sequences. **c.** Fold change of expression of Repbase L1Md families in *Dnmt1-/-* and DKO embryos as a function of their evolutionary age in million of years (Myr) (taken from Sookdeo et al., 2013). **d.** Density histogram of the log2(fold change) expression for the 4,593 individual TE copies significantly reactivated (fold change > 3, adjusted *p* value < 0.001) in *Dnmt1-/-* embryos. **e.** Pie chart showing the proportion of reactivated TE copies located in gene introns **f.** Pie chart showing the classification by family of the 4,593 individual TE copies reactivated in *Dnmt1-/-* embryos. **g.** RNA-seq tracks showing examples of derepressed ERV transposon copies in *Dnmt1-/-* embryos. Repeatmasker annotations are shown below the tracks.

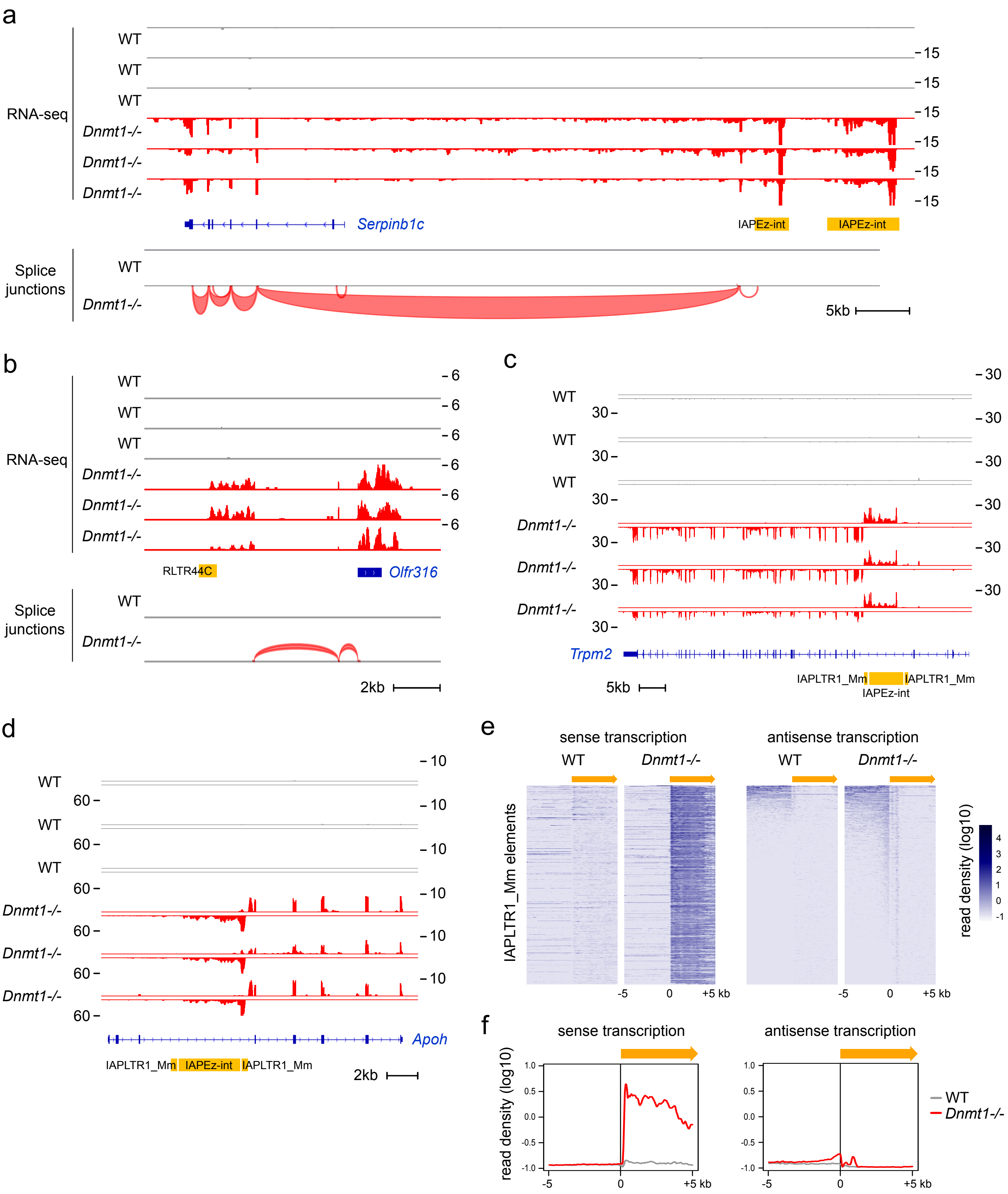


**Supplementary Figure 10. Derepressed ERVs modify the expression of proximal genes in *Dnmt1-/-* embryos. a-b.** *Serpinb1c* expression is induced by a long RNA, which initiates 40 kb upstream of the gene in a derepressed IAPEz element and splices into the exon 3. *Olfr316* expression is induced by an RNA initiating in an upstream RLTR44C element. The figures show RNA-seq tracks in WT and *Dnmt1-/-* embryos and splice junctions in one replicate of WT and *Dnmt1-/-* embryo. ERVs annotated by RepeatMasker are displayed in yellow. **c-d**. RNA-seq tracks of the *Trpm2* and *Apoh* genes in WT and *Dnmt1-/-* embryos. Internal initiation occurs from a derepressed intronic IAPLTR1_Mm located on the opposite strand. For each sample, the RNA-seq signal from the top and bottom strands are shown. IAPs annotated by RepeatMasker are displayed in yellow. **e.** Heatmaps of RNA-seq read density (log10) around IAPLTR1_Mm elements in sense and antisense orientation in WT and *Dnmt1-/-* embryos. For each genotype the heatmaps are sorted by total antisense signal. **f.** Metaplots showing the average RNA-seq signal around IAPLTR1_Mm elements in sense and antisense orientation in WT and *Dnmt1-/-* embryos. In e-f, the yellow arrows indicate the position and orientation of the IAPLTR1_Mm and their cognate IAPEz elements, and the values represent the mean of the three biological replicates.

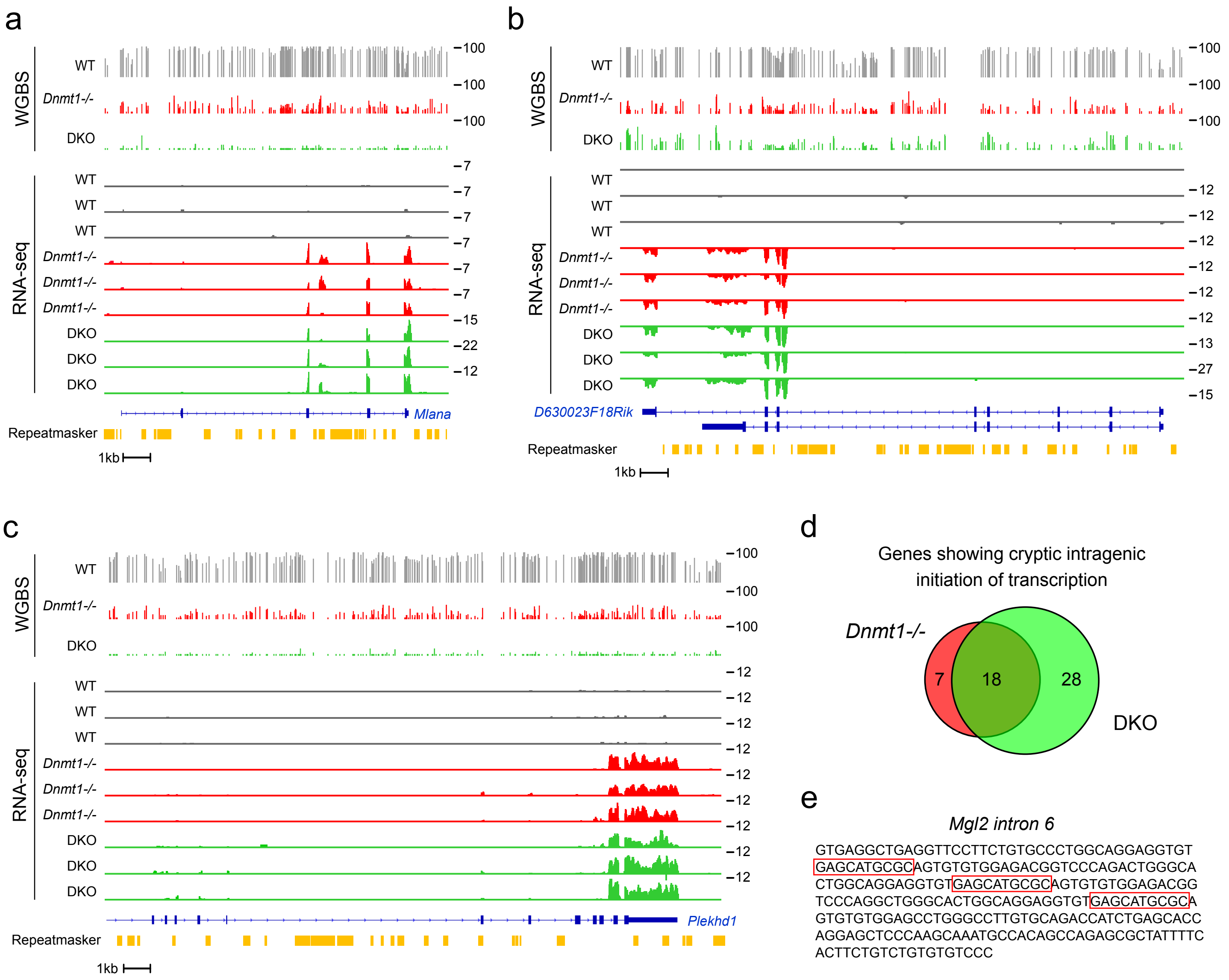


**Supplementary Figure 11. Cryptic transcription initiation from intragenic sequences in *Dnmt* mutant embryos. a-c.** Genome browser tracks of WGBS methylation profiles and RNA-seq for the *Mlana*, *Plekhd1* and *D630023F18Rik* genes in WT and *Dnmt* mutant embryos. One WGBS replicate and three replicates of RNA-seq are shown. The RepeatMasker annotation track is displayed below in yellow. **d.** Venn diagram showing the overlap between the lists of genes with cryptic intragenic initiation identified in *Dnmt1-/-* and DKO embryos. **e.** DNA sequence of the intron 6 in the *Mgl2* gene. DNA binding motifs of the transcription factor NRF1 are highlighted in red.
