# Supplementary material for: Genome-wide analysis in the mouse embryo reveals the importance of DNA methylation for transcription integrity
Source: Nat Commun. 2020 Jun 19;11:3153. doi: 10.1038/s41467-020-16919-w (PMC7305168; doi:10.1038/s41467-020-16919-w)
Supplement: Supplementary file 3 — Description of Additional Supplementary Files [file 41467_2020_16919_MOESM3_ESM.pdf]

Description of Additional Supplementary Files

Supplementary Data 1

Genomic coordinates and DNA methylation of imprinted germline DMRs in *Dnmt* mutant embryos.

Supplementary Data 2

Analysis of RefSeq gene expression (DESeq2) and genes with internal initiation in *Dnmt* mutant embryos.

Supplementary Data 3

Analysis of transposon expression in *Dnmt* mutant embryos.

Supplementary Data 4

Oligo sequences.
